# Granulomatous lobular mastitis in pregnancy: report of 29 cases

**DOI:** 10.3389/fonc.2025.1526754

**Published:** 2025-09-10

**Authors:** Hongyi Liang, Guangxi Shi, Hanhan Chen, Xiaofei Liu, Zhe Zhang, Aili Song, Jingwei Li

**Affiliations:** ^1^ The First Clinical Medical College, Shandong University of Traditional Chinese Medicine, Jinan, China; ^2^ Department of Breast and Thyroid Surgery, Affiliated Hospital of Shandong University of Traditional Chinese Medicine, Jinan, China

**Keywords:** granulomatous mastitis, pregnancy, etiology, manifestations, therapeutics, traditional Chinese medicine

## Abstract

Granulomatous lobular mastitis (GLM) is a rare inflammatory breast disease, and there are few reports of GLM in pregnancy (GLMIP). Therefore, this study retrospectively analyzed cases diagnosed with GLMIP from 2011 to 2023 and found that in patients with GLMIP there were varied demographic manifestations such as age, pregnancy weeks, and numbers of pregnancy and delivery, and several associated complications including erythema nodosum, arthritis and lower extremity edema. Among them, 82.8% of the patients received integrated traditional Chinese medicine (TCM) and western medicine treatment, and 17.2% of the patients received TCM treatment alone, but the application rate of TCM treatment was 100%. The results showed that both groups significantly improved the effective rate (91.7% and 60.0%, respectively), improved breast appearance (4.1% and 20.0%, respectively), reduced the rate of progression or recurrence rate (8.3% and 60.0%, respectively), and shortened the time for complete remission (13.793 months vs 12.625 months, respectively). To date this study is the one with the largest sample size of GLMIP, but also the one with the largest sample size in which the combination of TCM treatment and non-surgical treatment was applied. The complications include erythema nodosum, arthritis and lower extremity edema. Therefore, the application of TCM in the treatment of GLMIP is worth promoting vigorously.

## 1 Introduction

Granulomatous lobular mastitis (GLM), also known as idiopathic granulomatous mastitis, is a chronic inflammatory disease, with granuloma formation in the breast tissues being the major pathological feature (1). GLM was first reported in 1972 by Kessler et al. (2). GLM usually occurs in women of childbearing age, but the onset during pregnancy is extremely rare (3). According to clinical reports, the incidence of GLM in pregnancy (GLMIP) is less than 5.66% (4). However, the incidence of GLMIP has gradually increased in recent years. It has been reported that the etiology of GLM might be related to tuberculosis or other infection factors (5). The typical manifestations include breast nodules, masses, abscesses, and ulcers, with variations in different stages (6, 7). However, there was no specific laboratory and clinical examinations. Thus, GLM is often misdiagnosed as other breast diseases, such as breast cancer. Therefore, it is necessary to further explore the etiology and clinical manifestations of GLM during pregnancy so as to improve the diagnosis.

Currently, there is no standardized therapeutic protocol since etiology is unclear and clinical manifestations varies widely (8, 9). In clinic, surgical treatment and conservative treatment are common applied. Surgical treatment usually causes a large incision, and therefore affects breast cosmesis (10). Conservative treatment usually involves hormone therapy, steroid therapy and close observation. Hormone therapy and steroid therapy usually lead to various side effects, and therefore the recovery is slow in these patients under such therapies (11). Under close observation, patients usually take long time to recover (12).

Besides, during the pregnancy, there is more safety concerns for some drugs would not only affect the health of the pregnant women, but also the development of fetus. Sometimes, the incorrect administration of drugs would cause abortion or miscarriage (13).

Traditional Chinese medicine (TCM) treatment usually is of high safety without severe side effect, and therefore TCM treatment might be a safe and effective therapy for GLM (7, 14). A large number of clinical trials and lab experiments showed that internal and external treatment of TCM played an increasingly important role in the treatment of GLM (15, 16). According to these reports, it might be concluded that the integrated TCM and western medicine was effective for GLM. Since TCM treatment is with low recurrence rate and less breast deformity, TCM treatment might be more reasonable for GLM (17). Besides, under the guidance of TCM theory, there are many specialized TCM treatment modalities for GLM. It is gradually accepted by many researchers that the treatment by integrated TCM and western medicine is effective in shortening the disease course, reducing lesion scope, preserving breast cosmesis and reducing the recurrence rate of GLM (18, 19). However, there has been no consensus on the TCM therapeutic protocol.

Therefore, in this study, we retrospectively analyzed a series of cases diagnosed with GLMIP in our institution. The general data, clinical conditions, recurrence after treated with different treatment modalities, duration of treatment, and changes in breast appearance were analyzed. Besides, the risk factors, clinical manifestations, and the optimized treatment protocol were also summarized.

## 2 Materials and methods

### 2.1 Data source and diagnostic criteria

Female patients with GLM at the Department of Breast and Thyroid Surgery, The Affiliated Hospital of Shandong University of Traditional Chinese Medicine, from January 2011 to February 2021 were included in the study. The disease diagnostic criteria were the presence of histopathological features of acute and chronic granulomatous inflammatory abscesses on the lobules of the breast in pregnant women (20). This study was performed in accordance with the national regulations for human experimentation and approved by the Ethics Committee of the Shandong University of Traditional Chinese Medicine Affiliated Hospital (approval/permit number: LNJ-2022069-KY). Informed consent was obtained by the study participants prior to study commencement.

### 2.2 Inclusion and exclusion criteria

Inclusion criteria: (1) Patients with GLM attending the Department of Breast and Thyroid Surgery, The Affiliated Hospital of Shandong University of Traditional Chinese Medicine. (2) Patients who met the diagnostic criteria and the pathological diagnostic criteria for GLM. (3) Patients who could be followed up in outpatient clinic or by telephone.

Exclusion criteria: (1) Patients with indefinite pathological diagnosis or not meeting the diagnosis criteria of GLM. (2) Patients treated irregularly, or with incomplete information affecting statistical analysis, or failing to be followed up. (3) Patients with breast malignancy. (4) Patients with severe primary heart, liver, spleen, lung, kidney, hematological system diseases or psychiatric diseases.

### 2.3 Therapeutic methods

All patients were not treated with surgical excision. Patients with larger lesions were given catheter drainage. Besides, patients with positive results of pus bacterial cultivation were treated with corresponding antibiotics. Patients with lower extremity edema were treated with steroids. The duration of drainage, steroids and antibiotics treatment were 7-14 days.

All patients were treated with internal and external administration of Chinese medicinals. Fu Fang Huang Bai liquid (Chinese Drug Approval Number: No. Z10950097) was externally applied on the affected area, once per day. Fu Fang Huang Bai liquid was to clear away heat and detoxify including lian qiao (Forsythiae fructus), huang bai (Phellodendri chinensis cortectcm), jin yin hua (Lonicerae japonicae flos), pu gong ying (Taraxacl herba) and wu gong (Scolopendra). TCM formulas for internal administration were modified according to the condition of patients. Patients without fistula or ulcer were given Gua Lou Niu Bang decoction: gua lou (Trichosanthis fructus) 15 g, niu bang zi (Arctii fructus) 12 g, tian hua fen (Trichosanthis radix) 12 g, huang qin (Scutellariae radix) 12 g, zhi zi (Gardeniae fructus) 12 g, jin yin hua (Lonicerae japonicae flos) 12 g, lian qiao (Forsythiae fructus) 12 g, zao jiao ci (Gleditsiae spina) 12 g, qing pi (Citri reticulatae pericarpium viride) 6 g, chen pi (Citri reticulatae pericarpium) 6 g, chai hu (Bupleuri radix) 6 g and gan cao (Glycyrrhizae radix et rhizoma) 6 g. Patients with fistula or ulcer were given Tuo Li Xiao Du powder: ren shen (Ginseng radix et rhizoma) 6 g, huang qi (Astragali radix) 15 g, chuan xiong (Chuanxiong rhizoma) 3 g, dang gui (Angelicae sinensis radix) 10 g, bai shao (Paeoniae radix alba) 10 g, bai zhu (Atractylodis macrocephalae rhizoma) 10 g, jin yin hua (Lonicerae japonicae flos) 10 g, fu ling (Poria) 15 g, bai zhi (Angelicae dahuricae radix) 10 g, zao jiao ci (Gleditsiae spina) 10 g, gan cao (Glycyrrhizae radix et rhizoma) 5 g, and jie geng (Platycodonis radix) 10 g. The formulas were administered one dose per day. The medicinals were provided by Department of Chinese Medicinals, The Affiliated Hospital of Shandong University of Traditional Chinese Medicine. All patients took the above herbs until recovered.

### 2.4 Research methods

Clinical data were collected from 29 patients, including age, time of diagnosis, numbers of pregnancy weeks, pregnancies and deliveries, affected side, prolactin (PRL) level, erythrocyte sedimentation rate (ESR), C-reactive protein (CRP) level, results of bacterial culture of pus, clinical manifestations, systemic symptoms, previous history, treatment protocol, treatment effect and follow-up. The curative rate was determined by telephone follow-up 9 months after treatment to assess the efficacy. Patients were required to return to the hospital for ultrasound examination 6 months after the treatment to assess the recurrence rate.

### 2.5 Efficacy evaluation

#### 2.5.1 Criteria for efficacy evaluation

The evaluation criteria were established with reference to the 2018 edition of the Diagnostic Efficacy Criteria for Chinese Medical Conditions (21). Effective refers to that the mass shrinks or disappears, the redness, hot sensation and pain disappear, and the fistula disappears or nearly disappears, and ineffective refers to that mass does not shrink or disappear, and the fistula does not disappear. Efficacy rate is the ratio of the number of effective cases to the total number of cases 9 months after initiating the treatment.

#### 2.5.2 Changes in breast appearance

The evaluation criteria were the Harris evaluation criteria from Breast Plastic Surgery (22).

Harris evaluation criteria

Excellent: The treated breast is almost identical in size and shape to the contralateral breast.

Good: The retraction of the breast and/or skin changes involve less than 1/4 of the original.

Fair: The retraction of the breast and/or skin changes involves 1/2-1/4 of the original.

Poor: Deformity involves more than 1/2 of the breast.

The scar size was calculated by measuring the length and area.

The excellent and good were defined as no change in breast appearance. Fair and poor were defined as changes in breast appearance.

#### 2.5.3 Progression or recurrence rate

Six months after the treatment, patients were followed up to determine whether there was progression or recurrence. The progression or recurrence rate is the ratio of the number of progression or recurrence cases to the total number of cases.

#### 2.5.4 Complete remission time

The time of complete remission was determined through follow-up. The standard of complete remission was that the mass disappeared and the fistula healed completely.

### 2.6 Statistical analyses

Firstly, patient information and follow-up data were collated and recorded. Secondly, a patient database was established using Microsoft Excel 2019, and SPSS 25.0 (SPSS Inc., Chicago, IL) was used for statistical analysis. Measurement data were expressed as mean ± standard deviation (SD), and enumeration data were expressed as number or percentage of cases. W test was conducted for normality, and F test for homogeneity of variance. For the comparative analysis between the two groups, measurement data were tested by t test or Wilcoxon rank-sum test and enumeration data were compared by the chi-square test. P<0.05 was considered significant.

## 3 Results

### 3.1 Demographic characteristics and clinical manifestations

**Figure 1:**
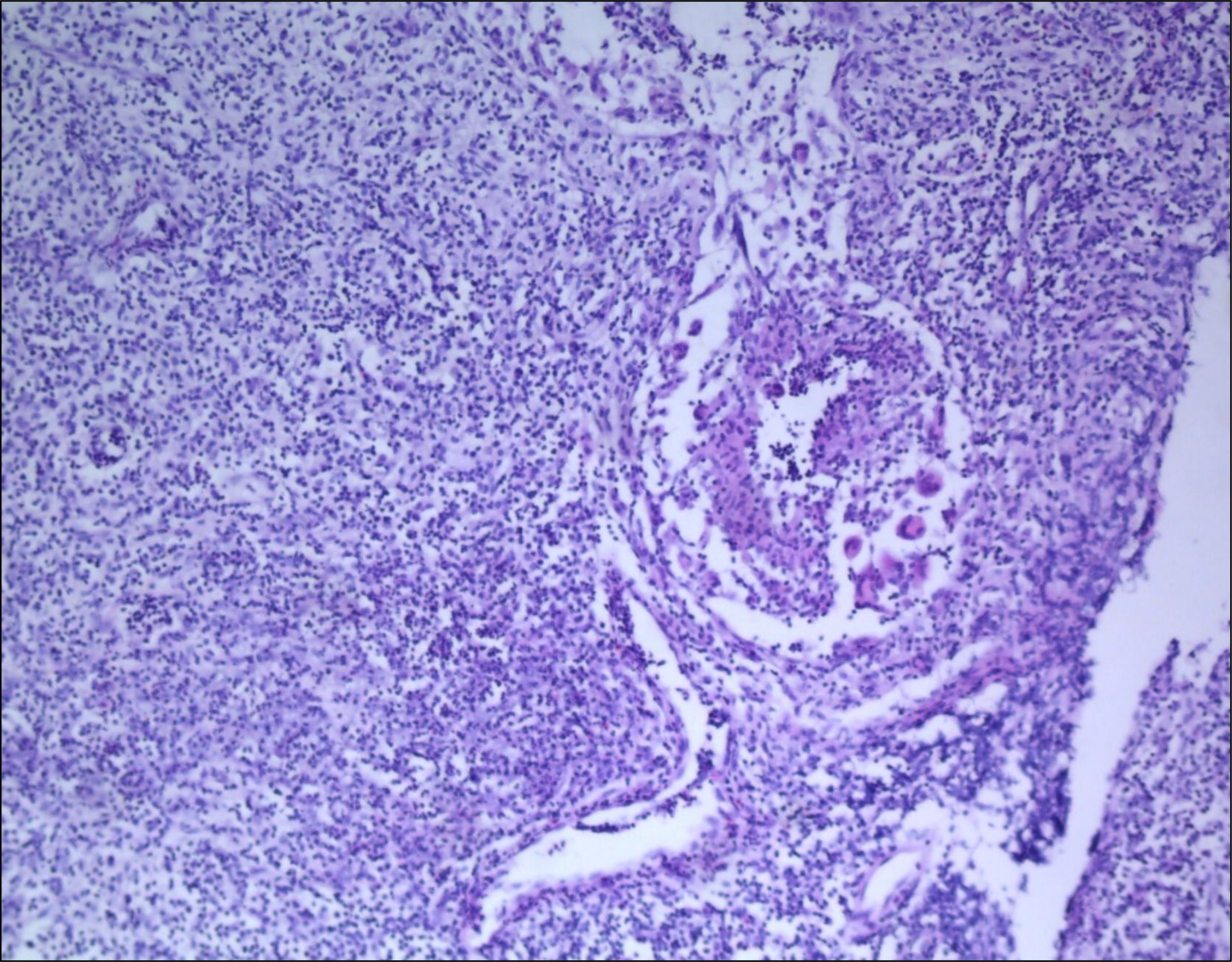
Typical pathological manifestations of GLMIP. Staining: H&E staining: 40×. (Credit: Department of Pathology, The Affiliated Hospital of Shandong University of Traditional Chinese Medicine).

**Figure 2:**
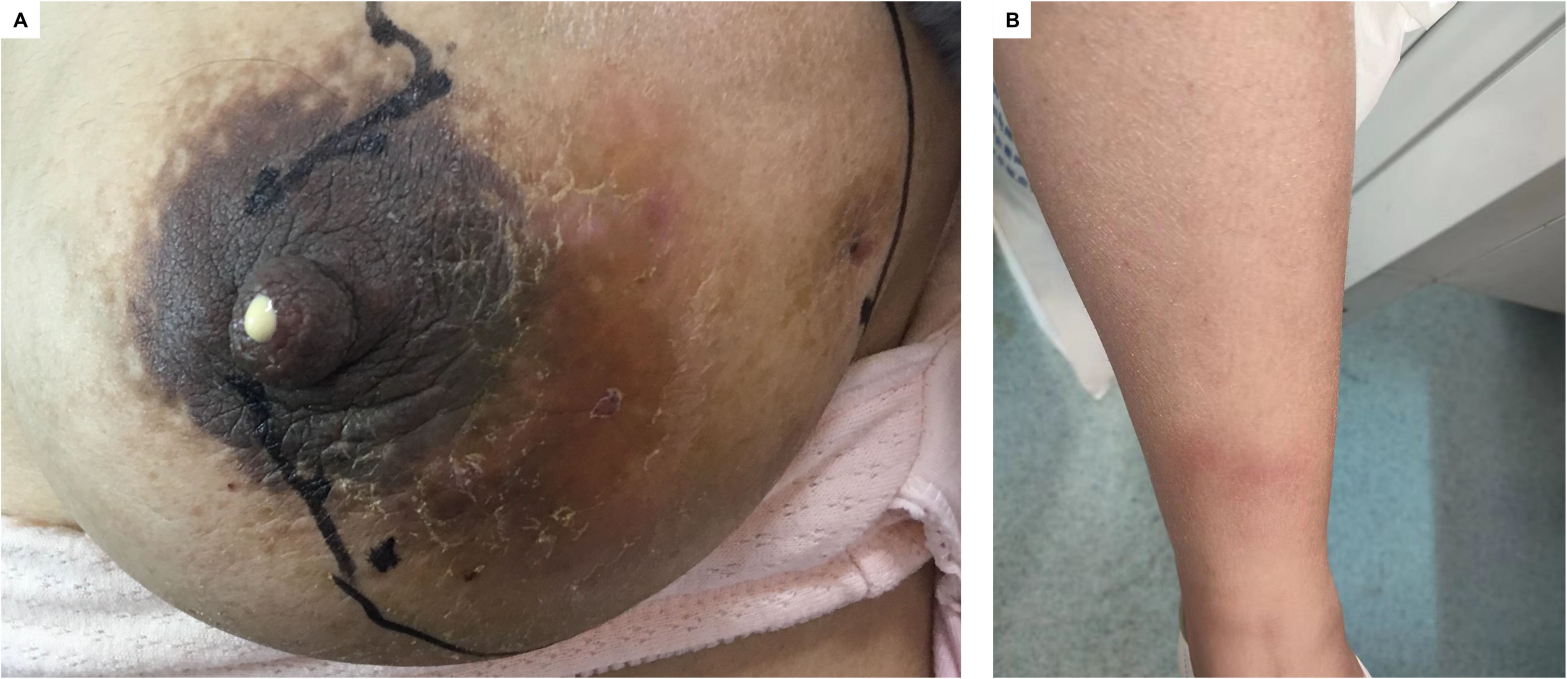
Typical clinical symptoms of GLMIP. **(A)** Extensive redness and swelling of the left breast **(B)** Erythema nodosum on the left lower extremity.

A total of 29 female patients were pathologically diagnosed with GLMIP over an 11-year period from 2011 to 2021 in our institution. Pathological examination showed there were vacuole or micro-abscess in the lesion center surrounded by layers of inflammatory cells including epithelioid cells, neutrophils, and scattered multinucleated giant cells (**Figure 1**). **Table 1** showed the basic demographic characteristics and clinical presentation of the patients, which included age at diagnosis, pregnancy weeks, number of pregnancy and delivery, affected side, PRL level, ESR level, CRP level, clinical features and systemic symptoms. The mean age of the patients at diagnosis was 30.448 years (21-38 years) and the mean pregnancy weeks was 21.172 weeks (5-39), 20 patients (70.0%) had one delivery, 4 patients (13.8%) had no delivery, 5 patients (17.2%) had two deliveries. All patients had at least one pregnancy, 11 patients (37.9%) had two pregnancies, 11 patients (37.9%) had three pregnancies, and 2 patients (6.9%) had four pregnancies. All patients had unilateral lesions. 16 patients (55.2%) had left-sided onset and 13 patients (44.8%) had right-sided onset. The level of PRL was higher than the normal in 20 patients (83.3%), ESR was higher than the normal in 21 patients (100%) and the level of CRP was higher than the normal in 13 patients (100%). Unfortunately, data about PRL, ESR and CRP could not be obtained from some patients. In 12 patients (41.4%) the results of bacterial culture of pus were positive. In all patients, breast lumps were present. In most patients there were redness (89.7%), hot sensation (82.8%), and pain (89.7%) of the skin, and in 4 patients (13.8%) there were both fistula and ulcer present, in 1 patient (3.4%) there were only fistula present and 2 patients (6.9%) only ulcer present. Of the systemic symptoms, in 10 patients (34.5%) there were nodular erythema present, in 11 patients (37.9%) arthritis present and in 9 patients (31.0%) lower extremity edema present. **Figure 2** demonstrated the typical clinical presentation and presentation of erythema nodosum. Therefore, we could know that patients with GLMIP showed varied demographic manifestations including age, gestational weeks, number of pregnancy and delivery, as well as varied clinical manifestations including nodular erythema, arthritis and lower extremity edema.

**Table 1:** Demographic and clinical characteristics of 29 patients with GLMIP.

Case	Age at diagnosis in years	Pregnancy weeks	Numbers of pregnancy	Numbers of delivery	Affected side	PRL (uIU/mL)	ESR (mm/hr)	CRP (mg/L)	Pus bacterial cultivation	Symptoms at diagnosis	Constitutional symptom
1	24	24	1	0	Right	–	–	–	Positive	ABCD	abc
2	24	24	1	0	Right	–	–	–	Negative	AB	None
3	33	16	2	1	Left	7230	–	–	Negative	ABCDEF	b
4	23	24	1	0	Right	–	–	–	Positive	ABCDF	ac
5	33	16	2	1	Left	3883	67	–	Negative	ABCD	b
6	37	24	2	1	Left	3794	56	–	Positive	ABCD	ac
7	29	26	2	1	Left	3443	53	15.0	Negative	ABCD	b
8	36	20	3	1	Right	1386	74	38.1	Negative	ABCDF	None
9	37	33	2	1	Left	3770	76	–	Positive	ABCD	ac
10	27	31	3	1	Right	6065	47	–	Negative	ABCD	c
11	37	35	3	1	Left	2987	85	43.9	Negative	ABCD	b
12	21	14	1	0	Left	–	66	27.3	Negative	AD	None
13	28	5	3	2	Right	992.6	75	65	Negative	ABCD	None
14	29	12	3	1	Left	1459	–	–	Positive	ABCD	ab
15	34	39	3	2	Left	359.9	68	–	Negative	AD	None
16	34	23	3	1	Left	4022	63	34	Positive	ABCDEF	None
17	24	33	2	1	Right	5592	78	–	Negative	ABCD	b
18	24	19	2	1	Left	7613	116	–	Positive	ABCD	ab
19	38	6	4	2	Right	244	–	3.78	Negative	AB	None
20	32	33	2	1	Left	>10000	104	–	Negative	ABCDEF	ab
21	32	17	3	1	Right	2801	52	33.2	Positive	ABCDE	ac
22	34	20	3	1	Left	2684	35	22.1	Negative	ABCD	c
23	35	8	2	1	Right	110	115	90.2	Negative	ABCD	None
24	33	5	3	2	Left	138.3	–	–	Positive	AB	ab
25	31	28	4	2	Right	5807	–	12.2	Positive	ABCDEF	None
26	33	16	2	1	Left	6554	32	11.8	Negative	ABCD	None
27	29	16	3	1	Left	–	80	26.7	Positive	ACD	ac
28	26	31	2	1	Right	3204	111	–	Negative	ABCD	b
29	26	16	2	1	Right	4309	99	–	Positive	ABCD	c

PRL, Prolactin; ESR, Erythrocyte sedimentation rate; CRP, C-reactive protein; A, Skin redness; B, Breast lump; C, Breast hot sensation; D, Pain Skin redness; E, Fistula, F, Ulcer; a, Erythema nodosum; b, Arthritis; c, Lower extremity edema.

### 3.2 Correlation analysis

**Figure 3:**
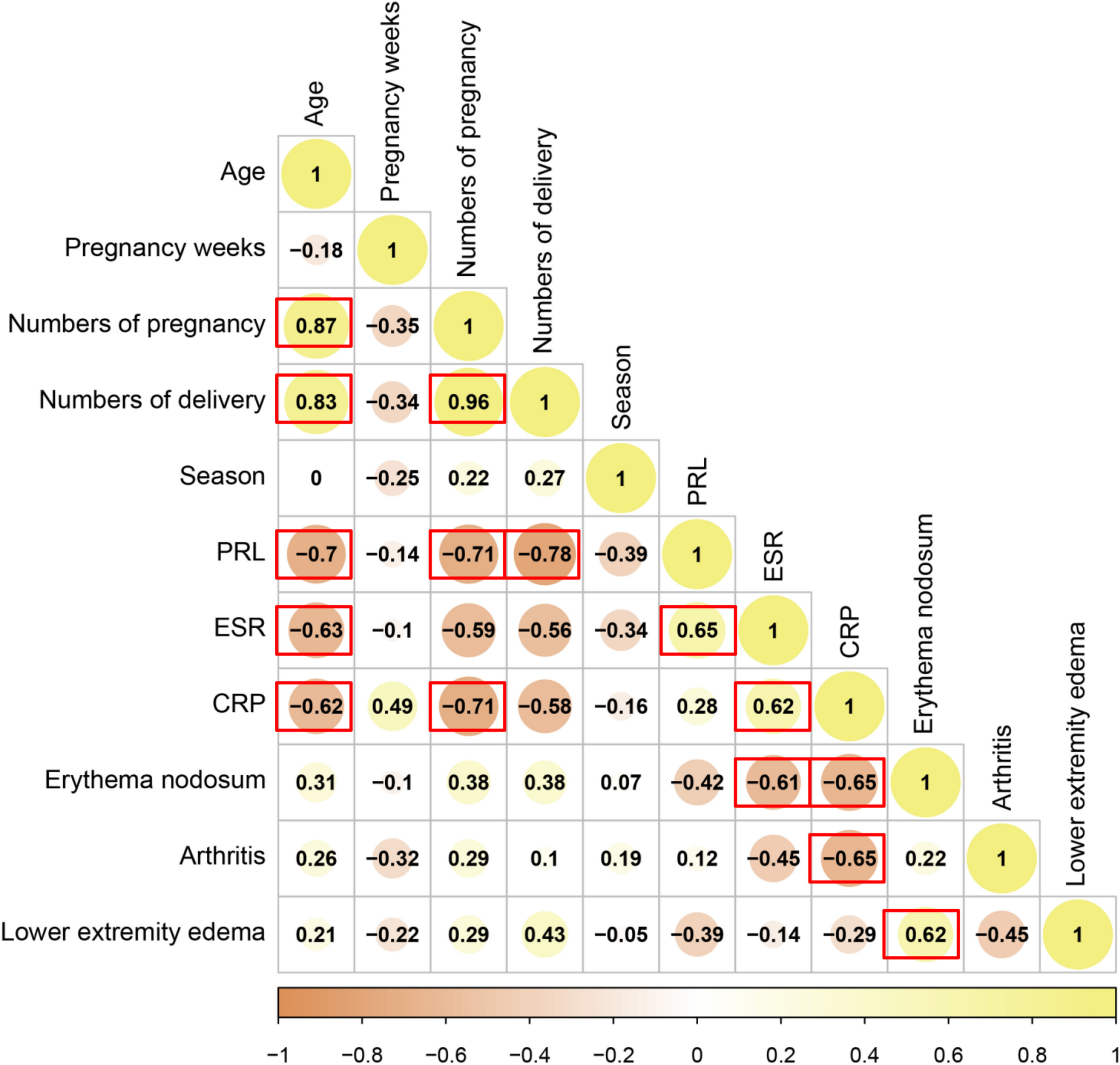
Correlation analysis of demographic characteristics. Rectangle with red border means P<0.05, statistically significant.

To further explore the correlation between demographic characteristics and clinical manifestations, correlation analysis was conducted. We found that there were significant correlations between patient age, pregnancy weeks, numbers of delivery, and levels of PRL (P<0.05), but also between levels of ESR and CRP, erythema nodosum and arthritis (P<0.05). **Figure 3** demonstrated the correlation analysis between demographic characteristics and clinical manifestations.

### 3.3 Etiological analysis

**Table 2** showed the risk factors and predisposing causes. GLM occurred in 12 patients (41.4%) in the seasons of spring, summer, and winter, while occurred in 17 patients (58.6%) in the season of fall. Of the 24 patients with PRL data obtained, hyperprolactinemia was present in 20 patients (83.3%). In 2 patients (6.7%) the occurrence of GLM was related to previous mastitis or abscess, in 3 patients (10.3%) related to breast trauma or smoking, and in 9 patients (31.0%) related to oral contraceptives or breast massage. However, most patients had history of inverted nipple (51.7%) or mammary tube blocking (75.9%). Therefore, season, hyperprolactinemia, and previous history of breast diseases might be the risk factors for GLM.

**Table 2:** Speculation on the related pathogenic factors of 29 cases of GLMIP.

Characteristics		N (%)
*Season*	Spring	6/29 (20.7%)
	Summer	4/29 (13.8%)
	Autumn	17/29 (58.6%)
	Winter	2/29 (6.7%)
*PRL (uIU/mL)*	>496	20/24 (83.3%)
	≤496	4/24 (16.7%)
*Inverted nipple*	Yes	15/29 (51.7%)
	No	14/29 (48.3%)
*Previous mastitis or abscess*	Yes	2/29 (6.7%)
	No	27/29 (93.1%)
*History of breast trauma*	Yes	3/29 (10.3%)
	No	26/29 (89.7%)
*History of oral contraceptives*	Yes	9/29 (31.0%)
	No	20/29 (69.0%)
*History of breast massage*	Yes	9/29 (31.0%)
	No	20/29 (69.0%)
*History of smoking*	Yes	3/29 (10.3%)
	No	26/29 (89.7%)
*History of mammary tube blocking*	Yes	22/29 (75.9%)
	No	7/29 (24.1%)

### 3.4 Treatment and prognosis

**Supplementary Table S1** showed the treatment modalities, outcomes and follow-up of 29 patients with GLMIP. All patients were treated with TCM (100.0%), 14 patients (48.3%) were treated with drainage, 10 patients (34.5%) were treated with steroids, and 12 patients (41.4%) were treated with antibiotics. Among all the patients, 4 patients (13.8%) received a combination of the four treatment modalities. After treatment, the majority (86.2%) of patients were in remission and only 4 patients (13.8%) were not in remission. The average follow-up time for all patients was 46.621 months (12-138 months). Only 5 patients (17.2%) showed progression or recurrence. In addition, the average time for complete remission in all patients was 13.793 months (9-27 months). The majority (93.1%) had no change in breast appearance. All patients gave birth to healthy babies. Therefore, we could conclude that in patients treated with TCM good therapeutic effect could be achieved.

We further analyzed the effects of integrated TCM and western medicine treatment and that of the TCM treatment alone. Among the 29 patients, all received TCM treatment (100.0%), with 5 patients (17.2%) receiving TCM treatment alone and 24 patients (82.8%) receiving TCM treatment combined with drainage and/or steroids and/or antibiotics. Before treatment, in terms of clinical manifestations and complications, both groups of patients had varying degrees of skin redness, breast lump, breast hot sensation, pain, fistula, ulcer, erythema nodosum, arthritis, and lower extremity edema. After treatment, there were significant improvements in the effective rate, breast appearance changes, progression or recurrence rate, and complete remission time in both groups of patients. Therefore, it could be concluded that the full course treatment of TCM played a key role in improving efficacy, improving breast appearance, reducing progression or recurrence, and shortening the time for complete remission. **Table 3** demonstrated therapeutic effects of integrated TCM and western medicine treatment and TCM alone. In addition, in this study, 24 patients (75.9%) t were given oral administration of Gua Lou Niu Bang decoction and 5 patients (24.1%) were given oral administration of Tuo Li Xiao Du powder. **Supplementary Table S2** listed the consisting medicinals of both formulas and the effects of them, respectively. Both formulas were with the function of detoxification and anti-inflammation. Patients without fistula or ulcer were given Gua Lou Niu Bang decoction, while patients with fistula or ulcer were given Tuo Li Xiao Du powder. We found that the recovery time was shortened and the breast cosmesis was little affected with the application of TCM treatment.

**Table 3:** Comparison of patients with GLMIP treated by TCM alone and integrated TCM and western medicine treatment.

			Integrated TCM and western medicine treatment (24)	TCM alone (5)
Baseline comparisons	Age at diagnosis (years)	30.448; SD 4.85 (n=29)	30.042; SD 5.10 n=24 (82.8%)	32.4; SD 2.58 n=5 (17.2%)
	Pregnancy weeks	21.172; SD 9.19 (n=29)	21.833; SD 9.09 n=24 (82.8%)	18.0; SD 9.01 n=5 (17.2%)
Treatment efficacy comparisons	Skin redness	26/29 (89.7%)	21/24 (87.5%)	5/5 (100%)
	Breast lump	29/29 (100%)	24/24 (100%)	5/5 (100%)
	Breast hot sensation	24/29 (82.8%)	19/24 (79.2%)	5/5 (100%)
	Pain	26/29 (89.7%)	21/24 (87.5%)	5/5 (100%)
	Fistula	5/29 (17.2%)	3/24 (12.5%)	2/5 (40.0%)
	Ulcer	6/29 (20.7%)	3/24 (12.5%)	3/5 (60.0%)
	Erythema nodosum	10/29 (34.5%)	9/24 (37.5%)	1/5 (20.0%)
	Arthritis	11/29 (37.9%)	9/24 (37.5%)	2/5 (40.0%)
	Lower extremity edema	9/29 (31.0%)	9/24 (37.5%)	0/5 (0)
	Effective rate	25/29 (86.2%)	22/24 (91.7%)	3/5 (60.0%)
	Changes in breast appearance	2/29 (6.9%)	1/24 (4.2%)	1/5 (20.0%)
	Progression or recurrence rate	5/29 (17.2%)	2/24 (8.3%)	3/5 (60.0%)
	Complete remission time	13.793; SD 4.10 (n=29)	12.625; SD 2.82 n=24 (82.8%)	19.4; SD 4.63 n=5 (17.2%)

Integrated TCM and western medicine treatment consists of drainage and/or steroids and/or antibiotics and TCM treatment; *SD*, standard deviation.

## 4 Discussion

GLM is a chronic inflammatory disease of breast tissue. It usually occurs in female reproductive period but is rare in pregnancy and lactation. The particularity and rarity of the disease increase the difficulty of distinguishing and treating. For GLM occurring during pregnancy, it is very likely to be misdiagnosed as malignant tumor, which could cause great psychological burden and delivery risk to pregnant women (23, 24). Therefore, it is important to understand the risk factors and clinical manifestations to avoid misdiagnosis. Besides, surgical resection therapy, which is widely applied in the treatment of GLM in recent years, might bring more risks in pregnant women. Therefore, the pathological characteristics of GLMIP should be explored to study the pathogenic factors, clinical manifestations and treatment methods. However, there are few reports of GLM cases during pregnancy in recent years.

In recent years, many studies have attempted to explain the mechanism of GLM from multiple factors such as immunology, endocrinology, and infectious diseases. Firstly, immune system dysfunction is considered one of the core causes of GLM. During pregnancy, a woman’s immune system undergoes significant adjustments to protect the fetus from maternal immune rejection. In this context, breast tissue may overreact to external or endogenous antigens, leading to granulomatous inflammation. Research has shown that there is a significant infiltration of macrophages, T cells, and other immune cells in tissue samples of GLM patients, suggesting that it is an immune-mediated pathological response (25). In addition, studies have found that the levels of cytokines (such as tumor necrosis factor alpha, interleukin, etc.) in the serum of GLM patients are significantly elevated, further supporting the correlation between immune dysfunction and the disease (26). Secondly, endocrine factors, especially hormonal changes during pregnancy, may be one of the triggers for GLM. During pregnancy, the levels of hormones such as estrogen and progesterone significantly increase, which not only leads to the proliferation and activation of breast tissue, but may also trigger local immune and inflammatory reactions through complex regulatory mechanisms (27). In addition, prolactin plays an important role in breast development and milk secretion, and abnormal fluctuations in prolactin levels may also be involved in the pathogenesis of GLM (28). On the other hand, although GLM is generally considered non-infectious, recent studies have begun to focus on the potential association between microbial infections and GLM (29). There is literature reporting evidence of bacterial and fungal infections detected in pathological samples of some GLM patients, suggesting that these microorganisms may act as triggering factors to activate the host’s immune system, leading to granuloma formation. However, the role of microbial infection in GLM remains controversial, and most studies still tend to consider GLM as a self-limiting, non-infectious inflammatory response (30). Although existing research has provided some clues to reveal its etiology, the exact mechanism of the disease still needs further exploration, and further analysis of clinical samples is particularly important.

This report is by far one with the largest sample size of GLMIP. By analyzing the demographic characteristics of the 29 patients, we found that hyperprolactinemia, inverted nipple, previous history of mastitis, abscess, breast trauma, or oral contraceptives, breast massage, smoking, and mammary tube blocking were the causes of morbidity. Our finding was consistent with the results of many previously published series. More importantly, we surprising found that GLM usually occurred in fall season. It was possible that during fall season, the environmental temperatures vary greatly, which would affect the human body negatively, and therefore chronic diseases tend to recur more often (31). Besides, among the main manifestations of GLM, breast lumps, pain, redness, and hot sensation occurs in most patients. The abscess could rupture and form a ductal fistula, thus manifesting as ulcers and fistulas. We also found that patients had 3 concomitant diseases, erythema nodosum, arthritis and edema of lower limbs, with a prevalence of 34.5%, 37.9% and 31.0%, respectively. Furthermore, levels of PRL and CRP, and ESR also increased. Relevant studies have proved that the increase of these indicators was closely related to erythema nodosum, arthritis and lower extremity edema (32). The reasons for the increase of these indicators might be that GLM is an autoimmune disease or be due to the physiological characteristics of pregnant women. Therefore, although the etiology of this disease was unclear and the clinical manifestations were unspecific, early prevention and treatment could be performed by understanding the risk factors for the occurrence and the series of symptoms of the disease.

Meanwhile, the treatment options regarding GLMIP were still a difficult problem to clarify. It had been reported that surgical excision, drainage, antibiotics, corticosteroids and clinical observation alone could be applied to treat GLM (33). Zuo et al. reported that pain in GLM could be relieved with surgical treatment, but the appearance of the breast was dramatically changed (34). Postolova et al. reported that the progression of the disease could be greatly controlled with corticosteroids and methotrexate, but disease recurrence was increased (35). Bouton et al. conducted observation without treatment, and they found that breast appearance was not affected but the disease progressed further (36). Although these treatments could achieve certain effects, there was no consensus on an effective treatment protocol. Moreover, since the patient was in pregnancy with weaker body, it is more difficult to treat.

TCM has been applied in treating mastitis since the ancient time, and recently TCM also has been applied for treating GLMIP for TCM treatment with the advantages of less side effects and non-invasive. According to the theory of TCM, on the one hand, the patient with GLM was of weak body, and therefore, the external evil qi (pathogenic factors) could easily invade the body and lead to the stagnation of evil toxins. On the other hand, due to the emotional anxiety of women during pregnancy, the liver qi becomes stagnant, with unsmooth circulation of qi, blood and body fluids, and finally there would be the heat generated by the stagnation. Eventually, the lumps that has already formed became abscesses, ulcers and even fistulas over time because of the heat generated, which caused more pain to pregnancy women. More importantly, during the pregnancy, many drugs are contraindicated. Improper use of drugs in early pregnancy may cause fetal death or malformation and improper use of drugs in middle and late pregnancy may cause brain and sexual organ malformation (37). Thus, TCM was very suitable for treating this disease.

This study is the one with the largest sample size in which the combination of TCM treatment and non-surgical treatment was applied to GLMIP. In this study, all 29 patients were not treated with surgical resection. In western medicine treatment there were only oral drugs and catheter drainage. All patients were treated with TCM internally and externally (38). Two classical formulas, Gua Lou Niu Bang decoction and Tuo Li Xiao Du powder, were for internal treatment through oral administration, with the function to clear heat and detoxify, relieve dampness and expel pus. Fu Fang Huang Bai liquid was applied for external treatment. Antibiotics, steroids, and catheter drainage were applied to treat local lesions in combination with TCM treatment in most patients (39). The results showed that there was no significant change in the appearance of the breasts in most patients, and the effective rate of a combination of TCM treatment was as high as 86.2%. The progression or recurrence rate of all patients was significantly reduced, and the time for complete recovery was also significantly shortened. This result indicated that a combination of TCM should be further promoted in clinical practice for the treatment of GLMIP.

However, there are some limitations of this study. Firstly, in terms of case inclusion, the cases included in this study were all from the same center, which may lead to bias in the diagnosis method of the disease with other centers. In addition, in terms of research methods, not only the pathogenesis was unclear but also there were no experiments *in vivo* and *in vitro*. Hence, more studies were needed to further clarify the underlying mechanism. Besides, in terms of research content, as a case study, this study has certain limitations in terms of data completeness and accuracy, and is prone to selection bias and recall bias, which also affects the reliability of the research results. In the future, management of included cases should be increased, and further prospective studies should be conducted to further increase the accuracy and reliability of the article. Furthermore, there was no control group in this study, which resulted in a lack of comparison and validation. In future studies, a non-TCM treatment control groups will be included to enhance the accuracy and persuasiveness of the research. Finally, the small size of the subgroups in this study reduces the statistical analysis ability of differences and the validity of conclusions is insufficient. Therefore, future studies need to increase the sample size of each group to increase the size of subgroup analysis and enhance the persuasiveness of statistical analysis.

In conclusion, GLM is a multifactorial and refractory disease with varied manifestations, and is rarer in pregnancy. Based on the complex etiology and clinical manifestations, a combination of TCM treatment could be applied to achieve better effect.

## 5 Conclusions

This study is the one with the largest sample size of GLM in pregnancy, and also the one with the largest sample size in which all patients were treated with TCM but without surgery. It could provide a novel protocol for treating GLMIP and should be further applied in clinic. GLMIP is a rare disease with multiple etiologies and varied manifestations, and it is refractory. The etiologies might be related to the season, inverted nipple, and mammary tube blocking. Complications may occur in parallel with erythema nodosum, arthritis, lower extremity edema. The combination of TCM for GLMIP can improve efficacy, improve breast appearance, reduce recurrence rate, and shorten the time to complete remission. Therefore, it should be further applied in clinic.

## Data Availability

The original contributions presented in the study are included in the article/**Supplementary Material**. Further inquiries can be directed to the corresponding author.

## Glossary

GLM: Granulomatous lobular mastitis
GLMIP: Granulomatous lobular mastitis in pregnancy
TCM: Traditional Chinese medicine
PRL: Prolactin
ESR: Erythrocyte sedimentation rate
CRP: C-reactive protein
SD: standard deviation.
