# Supplementary material for: Granulomatous lobular mastitis in pregnancy: report of 29 cases
Source: Front Oncol. 2025 Sep 10;15:1526754. doi: 10.3389/fonc.2025.1526754 (PMC12457945; doi:10.3389/fonc.2025.1526754)
Supplement: Supplementary file 1 [file Table1.docx]

**Table S1** Therapeutic effect and follow-up of 29 cases of GLMIP

| Case | Treatment regimen | Outcome | Follow-up (Month) | Progression or recurrence | Time to complete remission (M) | Changes in breast appearance | Growth and development of the newborn | |
| --- | --- | --- | --- | --- | --- | --- | --- | --- |
| 1 | ABCD | R | 138 | No | 13 | No | | Normal |
| 2 | AD | R | 120 | No | 11 | No | | Normal |
| 3 | D | R | 105 | No | 21 | No | | Normal |
| 4 | BCD | NR | 94 | No | 11 | No | | Normal |
| 5 | AD | R | 94 | No | 9 | Yes | | Normal |
| 6 | CD | R | 74 | No | 13 | No | | Normal |
| 7 | BD | R | 65 | No | 18 | No | | Normal |
| 8 | D | NR | 65 | No | 27 | No | | Normal |
| 9 | BCD | R | 52 | No | 12 | No | | Normal |
| 10 | ABD | R | 48 | Yes | 11 | No | | Normal |
| 11 | AD | R | 46 | No | 12 | No | | Normal |
| 12 | AD | R | 42 | No | 9 | No | | Normal |
| 13 | D | NR | 41 | Yes | 13 | No | | Normal |
| 14 | ACD | R | 34 | No | 11 | No | | Normal |
| 15 | AD | R | 32 | No | 16 | No | | Normal |
| 16 | CD | R | 29 | No | 16 | No | | Normal |
| 17 | AD | R | 28 | No | 17 | No | | Normal |
| 18 | CD | R | 28 | No | 11 | No | | Normal |
| 19 | AD | R | 28 | No | 9 | Yes | | Normal |
| 20 | D | R | 27 | Yes | 19 | No | | Normal |
| 21 | ABCD | R | 23 | No | 18 | No | | Normal |
| 22 | BD | R | 23 | No | 16 | No | | Normal |
| 23 | AD | R | 21 | Yes | 12 | No | | Normal |
| 24 | CD | R | 17 | No | 12 | No | | Normal |
| 25 | ACD | NR | 17 | No | 11 | No | | Normal |
| 26 | D | R | 17 | Yes | 17 | No | | Normal |
| 27 | ABCD | R | 17 | No | 9 | No | | Normal |
| 28 | AD | R | 15 | No | 15 | No | | Normal |
| 29 | BCD | R | 12 | No | 11 | No | | Normal |

TCM, Traditional Chinese medicine; R, remission; NR, not remission; A, Drainage; B, Steroids; C, Antibiotics; D, TCM

**Table S2** Two classical TCM formulas of 29 cases of GLMIP

| TCM formula name | Ingredients | Number of cases (%) | Average duration for prescription (weeks/visit) | Clinical manifestation | Average daily dose (g) | Effects | Reference |
| --- | --- | --- | --- | --- | --- | --- | --- |
| *Gua Lou Niu Bang* decoction | *gua lou (Trichosanthis fructus), niu bang zi (Arctii fructus), tian hua fen (Trichosanthis radix), huang qin (Scutellariae radix), zhi zi (Gardeniae fructus), jin yin hua (Lonicerae japonicae flos), lian qiao (Forsythiae fructus), zao jiao ci (Gleditsiae spina), qing pi (Citri reticulatae pericarpium viride), chen pi (Citri reticulatae pericarpium), chai hu (Bupleuri radix), gan cao (Glycyrrhizae radix et rhizoma)* | 22 (75.9%) | 12.6 | Skin redness, Mass, Fever, Pain | 10.25 | Soothe the liver, clear the stomach, clear the breast and reduce swelling | *The Golden mirror of medicine*, Qing dynasty, China |
| *Tuo Li Xiao Du* powder | *ren shen (Ginseng radix et rhizoma), huang qi (Astragali radix), chuan xiong (Chuanxiong rhizoma), dang gui (Angelicae sinensis radix), bai shao (Paeoniae radix alba), bai zhu (Atractylodis macrocephalae rhizoma), jin yin hua (Lonicerae japonicae flos), fu ling (Poria), bai zhi (Angelicae dahuricae radix), zao jiao ci (Gleditsiae spina), gan cao (Glycyrrhizae radix et rhizoma), jie geng (Platycodonis radix)* | 7 (24.1%) | 17.6 | Skin redness, Mass, Fever, Pain, Fistula, Ulcer | 6.90 | Replenish qi, clear away heat, support poison and penetrate pus | *Surgical Authentic*, Ming dynasty, China |
